# A 10-Year Analysis of Surgical Interventions Applied to Migrants: A Border Hospital Experience During the Syrian Civil War

**DOI:** 10.4274/TJAR.2025.252054

**Published:** 2025-10-14

**Authors:** Ergün Mendeş, Neşet Gümüşburun

**Affiliations:** 1 Koç University Faculty of Medicine Hospital, Department of Anaesthesiology and Reanimation, İstanbul, Türkiye; 2 Medical Park Tokat Hospital, Clinic of Obstetrics and Gynecology, Tokat, Türkiye; 3 İstanbul Aydın University Faculty of Medicine, Department of Obstetrics and Gynecology, İstanbul, Türkiye

**Keywords:** Border healthcare services, migration, refugees, surgical interventions, war-related trauma

## Abstract

**Objective:**

The Syrian civil war has resulted in one of the largest refugee movements globally, significantly impacting Türkiye due to its geographic proximity. Surgical care represents a critical yet often overlooked aspect of healthcare services required by displaced populations. This study aimed to evaluate the demographic characteristics and surgical procedures performed on migrant patients over a ten-year period at a secondary-level hospital located on Türkiye’s southern border.

**Methods:**

A retrospective cohort study was conducted at Kilis State Hospital between March 2010 and January 2020. Surgical procedures were categorized by department, patient nationality, and type of surgery. Patients operated under the “war code” were analyzed separately to identify conflict-related injury patterns.

**Results:**

A total of 52,978 surgical procedures were performed, with 41.76% involving Syrian patients. The mean age was 31.28±20.33 years, and male patients predominated, especially among the war-injured subgroup (91.59%). The most active surgical departments were orthopedics and traumatology (20.63%), gynecology and obstetrics (17.51%), and general surgery (15.67%). Among war-related surgeries, orthopedics, neurosurgery, and plastic surgery departments played major roles.

**Conclusions:**

This study highlights the high surgical demand among migrant populations in border regions, especially in conflict settings. Strengthening healthcare infrastructure, maintaining accurate surgical records, and implementing multidisciplinary approaches are essential for meeting these needs. Our findings can inform future policies aimed at improving surgical care for displaced populations.

**Main Points**

• Migration due to armed conflicts has led to a significant surgical burden in border regions.

• Orthopedics, gynecology, and general surgery departments carried the highest surgical load among migrants.

• War-related injuries predominantly affected young males, with orthopedic trauma being the most common.

• Strengthening healthcare infrastructure in border hospitals is essential for managing the surgical needs of displaced populations.

## Introduction

Unfortunately, ongoing wars around the world have rendered migration an inevitable phenomenon. As a result, the concept of migration remains a pressing global concern. Over the past two decades, international migration has risen significantly, with approximately 258 million international migrants reported in 2017.^1^ If this trend continues, the number of migrants is projected to exceed 400 million by the year 2050.^2^

Due to its geographical location, Türkiye has historically opened its doors and extended assistance to individuals forced to migrate, particularly in response to conflicts in the Middle East. In this context, the Syrian civil war, which has been ongoing since 2011, continues to impact Türkiye-especially its border provinces.^3^ Many war casualties were transferred to southern cities such as Hatay and Kilis. Furthermore, due to the prolonged instability in the region, Türkiye’s southern border has hosted not only the wounded but also a large number of Syrian migrants. According to United Nations data, Türkiye ranks among the top countries hosting the largest number of refugees, having accommodated more than 3.7 million asylum seekers to date.^4, 5^

In general, refugees and migrants-particularly in Türkiye, one of the countries most affected by migration-have faced numerous challenges related to nutrition, shelter, security, and access to healthcare services.^6, 7^ Among these vulnerable populations, women and children have been disproportionately affected due to the persistent lack of regional stability.^8^ Through its healthcare policies targeting migrants-including the “open-door” policy-Türkiye has become the leading provider of humanitarian aid, serving as a model for neighboring countries.^9^

Globally, it is estimated that approximately 3 million surgical procedures are performed on migrants each year.^10^ However, only a limited number of countries have conducted studies addressing the surgical needs of migrant populations.^11^ A review of the existing literature on Syrian migrant patients in Türkiye reveals a lack of studies focusing specifically on the utilization of surgical services within the healthcare system.

Despite the high number of migrants receiving healthcare services in Türkiye, particularly along the southern border, the surgical needs and utilization patterns of this population remain largely undocumented. We hypothesize that a substantial proportion of surgical interventions in border-region hospitals are associated with migrant populations, reflecting the health burden of conflict-related displacement. Therefore, the aim of our study is to evaluate the surgical procedures performed on migrant patients, along with their demographic, and clinical characteristics, over a ten-year period at a secondary-level hospital located along Türkiye’s southern border. By doing so, we seek to address a critical gap in the literature and contribute data that may inform healthcare planning for displaced populations.

## Methods

This study was designed as a retrospective and observational cohort study and was conducted at Kilis State Hospital, a secondary-level healthcare facility located near Türkiye’s southern border with Syria. Throughout the study period, the hospital played a central role in providing surgical services both to the local Turkish population and to Syrian migrants displaced by the ongoing war in Syria. The study period covered ten years, from March 2010 to January 2020, thus encompassing both pre-conflict and conflict-related migration dynamics. Ethical approval for the study was obtained from the Ethics Committee of Gaziantep University Faculty of Medicine (approval no: 2019/486, date: 05.02.2020). All procedures involving human participants were conducted in accordance with the ethical standards of the Declaration of Helsinki and its later amendments. The requirement for individual informed consent was waived by the Ethics Committee due to the retrospective nature of the study.

Patient data were retrospectively obtained from the hospital’s electronic medical record system. All surgical procedures recorded in the operating theater system were reviewed. To maintain data homogeneity, minor interventions performed in the emergency department or outpatient clinics (e.g., superficial wound closure, minor drainage procedures) were excluded. Duplicate records were identified using patients’ temporary or permanent identification numbers and were removed from the dataset. Patients with complete information on age, gender, and nationality/ethnicity were included, whereas records with missing data were excluded from the analysis.

For each patient, demographic variables including age (in years), gender (male or female), and nationality/ethnicity (Turkish or Syrian) were recorded. The surgical department performing the operation and the type of procedure were also documented. Wherever possible, procedures were categorized as emergency or elective surgeries based on clinical records. Patients who underwent surgery for conflict-related injuries were identified in the system under the classification “war code” and were analyzed separately.

All surgical procedures were grouped according to the surgical department responsible for the operation. The departments included orthopedics and traumatology, gynecology and obstetrics, general surgery, urology, ear, nose and throat (ENT) surgery, paediatric surgery, plastic and reconstructive surgery, neurosurgery, ophthalmology, cardiovascular surgery, and thoracic surgery. Within each department, the most frequently performed specific procedures [e.g., cesarean section (C/S), appendectomy, orthopedic fixation surgeries, cataract surgery] were also analyzed.

Patients recorded under the “war code” classification were analyzed separately. The intensity of surgical activity by department, the predominant types of trauma, and demographic characteristics (age, gender) were evaluated specifically in this subgroup. Descriptive statistical methods were applied, and the results were presented as frequencies and percentages.

Among patients managed under the war code, injuries were classified into two main categories based on the mechanism. Direct war injuries resulting directly from combat-related causes such as gunshot, explosive, and shrapnel injuries. Indirect war injuries refer to trauma resulting from secondary effects of the conflict environment, such as being trapped under rubble, injuries sustained during evacuation, or secondary infections. All injuries resulting from either direct or indirect mechanisms were defined collectively as war trauma.

One of the major strengths of the study is that it covers a large patient population over an extended time period in a border region heavily affected by migration, providing a detailed overview of a wide surgical spectrum. However, the study’s single-center nature may limit the generalizability of its findings. Furthermore, the retrospective design carries the inherent risk of record errors or missing data, and these limitations were considered when interpreting the study results.

Regarding data security, all patient information was securely stored within the hospital’s encrypted electronic record systems, accessible only by authorized research personnel. Data were anonymized prior to analysis to prevent the disclosure of personal identifiers. Patient confidentiality and data protection principles were strictly adhered to throughout the study. The surgical departments performing the operations were grouped, and the types of procedures performed by each department were identified. Minor interventions performed outside the operating room were excluded from the study. Surgical diagnoses were evaluated based on the clinical departments where the patients were admitted and were subsequently referred to the operating room.

### Statistical Analysis

Descriptive statistical analyses were performed using IBM SPSS Statistics for Mac OS, version 27.0 (IBM Corp., Armonk, NY, USA). Categorical variables (such as gender, surgical department, type of trauma) were summarized as frequencies and percentages [n (%)], while continuous variables (such as age) were evaluated for normality through both visual (histograms, Q-Q plots) and analytical methods (Kolmogorov-Smirnov and Shapiro-Wilk tests) and reported as mean ± standard deviation. No data imputation was performed for missing data; incomplete records were excluded from the analysis.

To demonstrate the trends in surgical services over the years, annual surgical volumes by department and ethnicity were visualized using line charts, and the distribution of surgeries among Turkish and Syrian patients was presented in tabular format. A time-series (trend) analysis was used to assess changes in surgical service demands over time. A two-sided p-value of <0.05 was considered statistically significant for comparative analyses. However, as the primary aim of the study was descriptive analysis, inferential statistics were used in a limited scope.

## Results

A total of 52,978 surgical procedures were performed over the 10-year period examined at Kilis State Hospital. Of the operated patients, 30,854 (58.24%) were Turkish citizens, while 22,124 (41.76%) were of Syrian nationality. Regarding gender distribution, 31,201 (59.89%) were male and 21,777 (41.11%) were female. Among the Syrian patients, 13,750 (62.15%) were male and 8,374 (37.85%) were female.

The overall mean age of the patients was 31.28±20.33 years. The mean age of Turkish patients was 32.98±21.42 years, while the mean age of Syrian patients was 28.90±18.45 years. The overall mean age of the male patients was 29.04±20.78 years. The mean age of Turkish patients was 30.12±22.39 years, while the mean age of Syrian patients was 27.67±18.44 years. The overall mean age of the female patients was 34.48±19.23 years. The mean age of Turkish patients was 36.71±19.46 years, while the mean age of Syrian patients was 30.92±18.29 years.

When categorized by age groups, 12,454 patients (23.51%) were between 0 and 17 years, 28,030 (52.91%) between 18 and 44 years, 8,225 (15.53%) between 45 and 65 years, and 4,269 (8.06%) were over 65 years of age. Among the Syrian patients, these numbers were 5,650 (25.54%), 12,434 (56.20%), 2,903 (13.12%), and 1,137 (5.14%), respectively.

The age, gender, and ethnicity distribution of the patients included in the study are presented in Table 1.

### Distribution of Surgical Procedures by Ethnicity and Department

**Figure 1:**
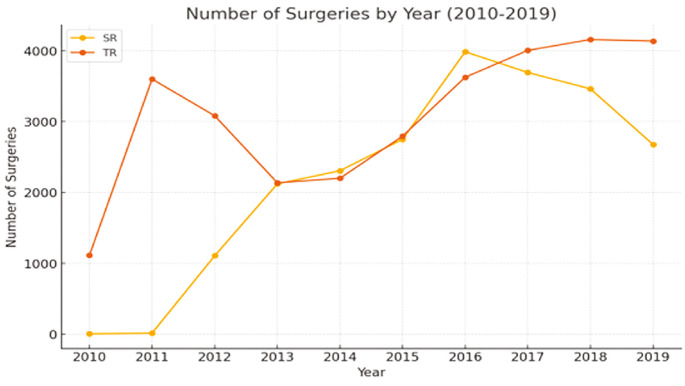
Number of surgeries by year. SR, Syrian; TR, Türkiye

Over the 10-year study period, the distribution of surgical procedures among Syrian and Turkish patients was analyzed across different surgical departments. The findings reveal considerable variations in service utilization patterns between the two groups. The change in surgeries performed on patients over the years is shown in Figure 1. The distribution of surgical clinics where patients received a service in the last 10 years by ethnic origin is shown in Table 2.

**Orthopedics and Traumatology:** A total of 10,929 procedures (20.63% of all surgeries) were performed in the orthopedics and traumatology department. Of these, 5,382 (10.16%) were Syrian and 5,547 (10.47%) were Turkish patients. Among Syrian patients, 2,500 surgeries (22.87%) were large bone osteotomy and fixation procedures, while 2,882 (26.37%) consisted of other orthopedic interventions. Turkish patients had a slightly higher proportion of miscellaneous orthopedic surgeries (33.59%) compared to fixation procedures (17.17%).

**Figure 2:**
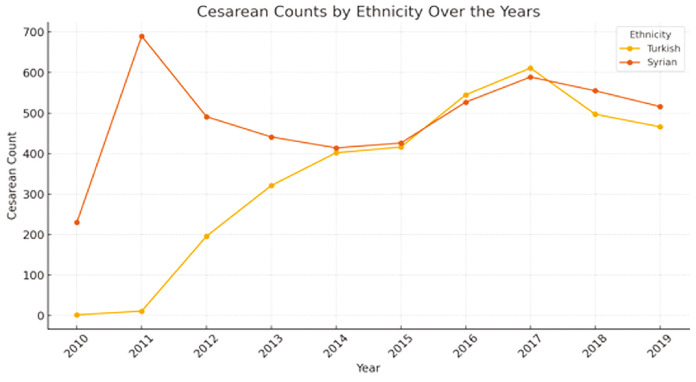
Cesarean counts by ethnicity over the years.

**Gynecology and Obstetrics:** The gynecology and obstetrics department accounted for 9,274 operations (17.51%). Of these, 3,786 (7.15%) were Syrian and 5,488 (10.36%) were Turkish. The majority of procedures in this department were C/S's, representing 90.26% of all obstetric surgeries. Specifically, 3,483 Syrian patients (37.56%) and 4,888 Turkish patients (52.70%) underwent C/S's. Other gynecologic surgeries were relatively less, making up 3.27% of their Syrian and 6.47% of their Turkish operations in this department. It has been observed that the number of births to Syrian patients has, over the years, caught up with and even exceeded the number of births to Turkish patients (Figure 2).

**General Surgery:** In the general surgery department, a total of 8,302 procedures (15.67%) were recorded-2,200 (4.15%) in Syrian and 6,102 (11.52%) in Turkish patients. While appendectomies were more frequent in Turkish patients (11.29%) than in Syrians (3.21%), emergency surgeries other than appendectomies were more common in Syrians (7.17%) than in Turkish patients (3.84%). Non-emergency surgeries dominated the overall caseload, especially among Turkish patients (58.37%).

**Urology:** The urology department performed 4,714 surgeries (8.90%), including 1,467 (2.77%) in Syrian patients, and 3,247 (6.13%) in Turkish patients. Turkish patients underwent circumcision most frequently (n = 959, 20.34%), whereas Syrian patients most commonly received endoscopic interventions (n = 620, 13.15%). Other frequently performed procedures included varicocele, hydrocele, and inguinal/testicular surgeries.

**Ear, Nose, and Throat (ENT):** A total of 4,642 surgeries (8.76%) were carried out in ENT, with 1,540 (2.91%) in Syrian and 3,102 (5.85%) in Turkish patients. The most frequent procedures were tonsillectomy and adenoidectomy, accounting for 18.50% of ENT surgeries in Syrians and 21.50% in Turkish patients. Septoplasty and rhinoplasty were also common, particularly among Turkish patients (26.84%).

**Paediatric Surgery:** In the paediatric surgery department, 4,130 operations (7.80%) were conducted-1,732 (3.27%) in Syrian and 2,398 (4.53%) in Turkish patients. Circumcision was the most common procedure among Turkish children, (n = 1,103, 26.70%), whereas non-emergency surgeries other than circumcision were more frequent in Syrian children, (n = 1,153, 27.92%). Emergency surgeries (excluding appendectomy) were also more common in Syrians.

**Plastic and Reconstructive Surgery:** A total of 3,151 operations (5.95%) were performed in this department-1,530 (2.89%) in Syrian patients and 1,621 (3.06%) in Turkish patients. Syrian patients underwent more skin and soft tissue surgeries (22.22% of the total surgeries) and maxillofacial procedures (8.31% of the total surgeries) compared to their Turkish counterparts. However, Turkish patients had a higher proportion of surgeries categorized as “others” (33.83%).

**Neurosurgery:** The neurosurgery department performed 2,893 surgeries (5.46%), with a notable ethnic distribution: 1,870 (3.53%) Syrian and 1,023 (1.93%) Turkish patients. Emergency neurosurgical procedures were significantly more common among Syrian patients, (n = 1,138, 39.34%) compared to Turkish patients, (n = 239, 8.26%).

**Ophthalmology:** There were 2,804 eye surgeries (5.29%) recorded-1,380 (2.60%) in Syrian and 1,424 (2.69%) in Turkish patients. Non-emergency procedures predominated for both groups, especially among Turkish patients (48.93%), indicating that this type of procedure constituted nearly half of the cases.

**Cardiovascular Surgery:** This department accounted for 1,670 surgeries (3.15%), of which 864 (1.63%) were Syrian and 806 (1.52%) were Turkish. Emergency interventions were more frequent among Syrian patients (33.71%), while Turkish patients had a greater proportion of non-emergency cardiovascular surgeries (40.31%).

**Figure 3:**
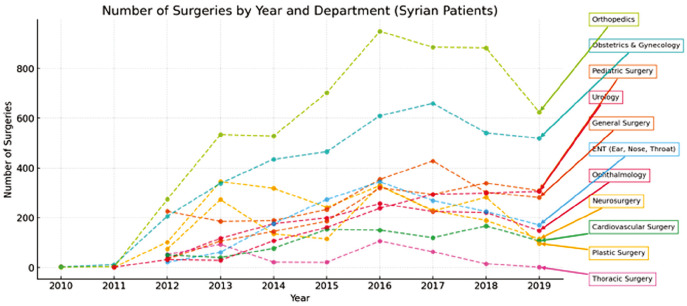
Number of surgeries by year and department (Syrian patients).

**Figure 4:**
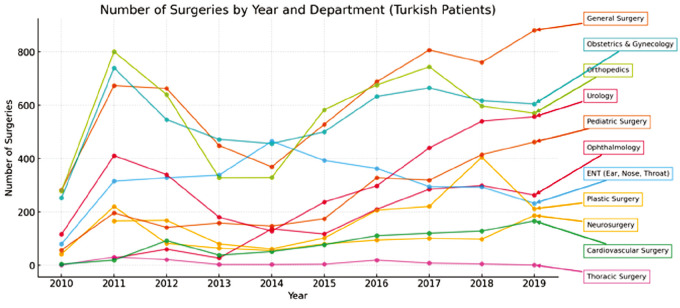
Number of surgeries by year and department (Turkish patients).

**Figure 5:**
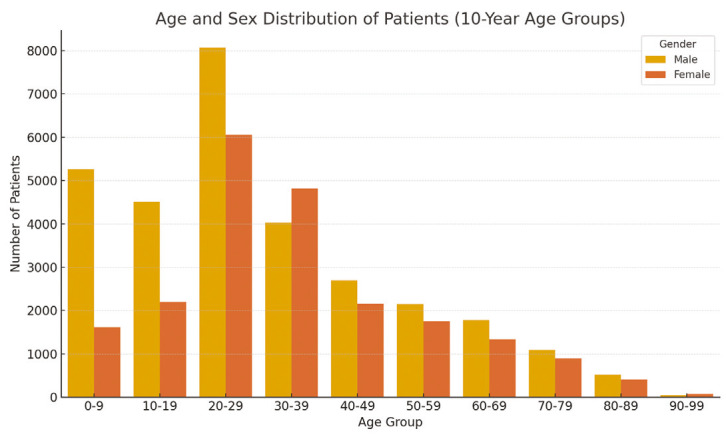
Age and sex distribution of patients (10-year age groups).

**Figure 6:**
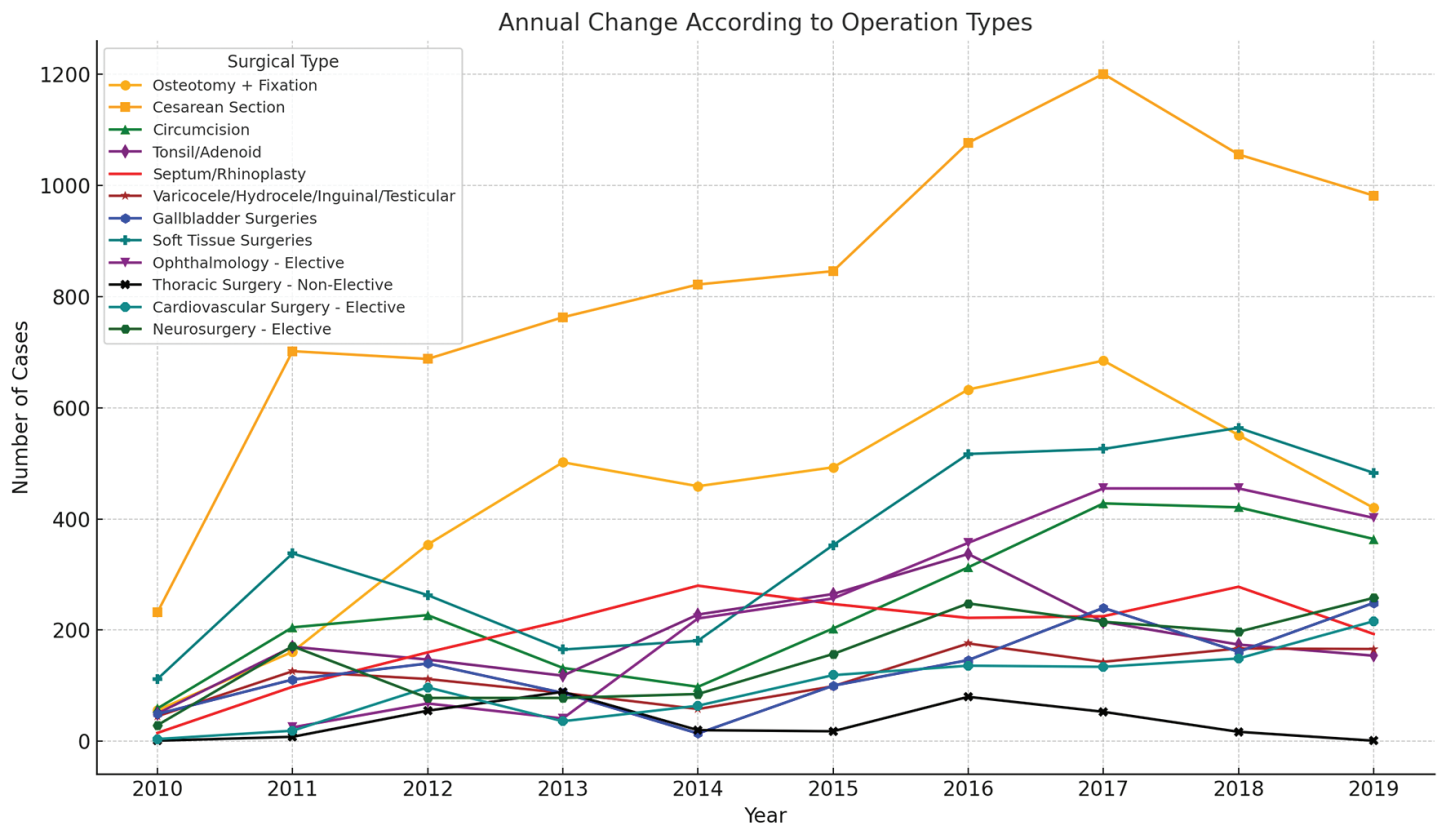
Annual change according to operation types.

**Thoracic Surgery:** Thoracic procedures were the least common, with 469 total operations (0.88%)-373 (0.70%) in Syrian and 96 (0.18%) in Turkish patients. Emergency thoracic surgeries were particularly high among Syrians (65.46%), indicating possible trauma-related indications (Figures 3, 4, 5, 6).

### Surgical Distribution of Refugees Operated Under “War Code”

Among the migrant patients operated under the “war code” classification, a total of 5,061 surgical procedures were performed across various departments. The distribution of these surgeries by surgical specialty is detailed below. Table 3 shows the surgical distribution of immigrants who were operated on under the war code.

The orthopedics and traumatology department performed the highest number of war-related surgeries, accounting for 1,739 procedures, which represented 34.70% of all operations in this category. These were predominantly related to trauma and fracture management resulting from conflict-related injuries. The second highest surgical load was observed in the neurosurgery department, with 811 procedures (16.18%), reflecting the frequency of head injuries and central nervous system trauma among war-injured patients. Plastic and reconstructive surgery ranked third, performing 792 operations (15.81%). These procedures were primarily related to soft tissue repair, burn injuries, and facial trauma commonly seen in war contexts. The general surgery department conducted 474 surgeries (9.46%), many of which likely involved emergency abdominal interventions due to penetrating or blunt trauma. Following this, cardiovascular surgery accounted for 403 interventions (8.04%), underscoring the complexity and severity of vascular injuries seen in war-wounded patients. The ophthalmology department performed 353 surgeries (7.04%), likely addressing globe injuries, orbital trauma, and other ocular complications caused by shrapnel or blast injuries. Thoracic surgery was involved in 262 cases (5.23%), frequently managing thoracic trauma such as rib fractures, hemothorax, or lung lacerations. Paediatric surgery accounted for 99 cases (1.98%), which, though less frequent, still indicates a concerning burden of war-related trauma among children. ENT surgeries were conducted in 50 cases (1.00%), and urology surgeries were conducted in 26 cases (0.52%), likely in response to complex trauma involving the genitourinary tract or head and neck. The gynecology and obstetrics department was the least represented, with only 2 procedures (0.04%), reflecting the trauma-focused nature of war-related surgical needs.

### Demographic Characteristics of Patients Operated Under the “War Code”

Among the patients who underwent surgery due to war-related injuries, 91.59% were male and 8.41% were female. The overall mean age of this patient group was 26.45 years. When stratified by gender, the mean age was 26.72 years in males and 23.51 years in females.

In terms of age distribution, 17.84% of the patients were under the age of 18, indicating a notable proportion of paediatric patients affected by war-related trauma. These findings reflect the predominantly young and male profile of individuals exposed to and injured by conflict conditions requiring surgical intervention.

## Discussion

Syria is one of the top three countries contributing most significantly to the global refugee population, and due to its geographic location, Türkiye is the country with the highest potential to host these refugees.^12^ Following the outbreak of the civil war in 2011, the Syrian healthcare system suffered severe infrastructural damage, rendering it incapable of providing even basic medical services. Consequently, mortality rates in Syria have increased steadily since that year.^13^ As a result, a large number of wounded individuals with high mortality risk were transferred to Türkiye, specifically to our hospital, for both surgical intervention and ongoing care.^14^ Our hospital, located along Türkiye’s southern border, has been significantly affected by the waves of migration since the onset of the Syrian civil war.^15^ More than 90% of Syrian refugees living in border camps and approximately 60% of those residing outside the camps, have utilized Turkish healthcare services.^16^ Nationwide, an estimated 1.5 million surgical procedures were performed on Syrian refugees during this period.^1^ In our hospital, numerous Syrian patients-both from within the province and via cross-border referrals–received surgical treatment.

Over this 10-year period, both Turkish and Syrian patients received equitable access to surgical care in our facility, without any significant differences in the quality or extent of services provided. We attribute this to our hospital being the only center in the province capable of performing surgical procedures. In line with previous studies, our findings confirm that healthcare facilities in border regions serve as the primary providers of medical services for migrant populations.^15, 17^ Moreover, contrary to the findings of Khalifeh et al.,^18^ our study revealed that in our region, migrants received surgical healthcare services to an extent equal to that of the local population. We believe this reflects not only the capacity of the Turkish healthcare system to provide high-quality care to Syrian migrants but also reflects a model that could guide other host countries in the region.

A review of the literature reveals that war affects not only combatants but also civilians. One study reported that 12% of war victims were children, while another indicated that 15% of children were affected by conflict.^19, 20^ In a study evaluating war victims based on age, the median age was reported as 19 years.^21^ In contrast, Aygün et al.^22^ reported a median age of 12.7 years, whereas Babacan et al.^1^ documented a median age of 28 years. We believe that these discrepancies in age distribution may be attributed to differences in study design. In our study, the overall mean age of patients was 31.28±20.33 years, which is higher than the average age of Syrian patients (28.90±18.45 years). Additionally, the mean age of Syrian migrants who underwent surgery under the war code was found to be even younger: 26.45 years. This may reflect the fact that younger individuals are more likely to be displaced during armed conflicts, whereas older adults may be less mobile.

A review of the literature suggests that the gender distribution of migrant patients presenting to hospitals is generally balanced.^1, 21, 22, 23^ However, in contrast to these findings, studies by Ağadayı et al.^24^ and Tahirbegolli et al.^21^ reported that male individuals were disproportionately more affected. In line with these latter findings, our study also revealed a statistically significant male predominance among Syrian migrant patients who underwent surgical procedures in our hospital, with 62.15% of the Syrian cohort being male. Among the war code-operated patients, the male proportion was even higher at 91.59%.

We believe that the primary reason for the observed male predominance among surgically treated Syrian migrants is that our hospital is the closest healthcare facility to both the border camps and active conflict zones. Consequently, many of the injured individuals were brought directly to our hospital for treatment. In addition, due to ongoing security concerns in the region, many migrants have settled permanently near the border, leading to a gradual increase in the number of female patients receiving surgical care over the years. Our findings demonstrated that gynecology and obstetrics emerged as the second most active surgical department, reflecting this shift. Notably, during this period, approximately half of the births in our hospital were among Syrian nationals, and in the later years of the study, the number of births among Syrian women surpassed those of Turkish women’s.

One study reported that the pregnancy prevalence among women of reproductive age in migrant populations ranges between 6% and 14%.^10^ Another study indicated that approximately 375,394 births occurred among Syrian migrants in Türkiye.^1^ Erdoğan and Çorabatır ^25^ projected that by 2025, around 1.8 million Syrian babies will have been born in Türkiye. Similarly, Özkılıç et al.^26^ observed a steady increase in the number of births among Syrian migrants. Consistent with these reports, our study revealed that the number of births among migrant women surpassed that of Turkish women over the study period.

In contrast to the findings of Ibrahim et al.,^27^ we observed a decline in C/S rates among Syrian patients in our region. In our previous study, we attributed this trend to the cultural preference of migrants for vaginal delivery over C / S.^28^ Globally, it has been reported that approximately 10% of the minimum essential surgical needs are related to obstetric complications, including cesarean delivery, postpartum hemorrhage, and ectopic pregnancy.^10^ Furthermore, two previous studies reported that maternal-fetal obstetric procedures accounted for 17% to 43% of all surgical interventions.^29, 30^ Our study supports these findings by showing that even during periods of intense conflict, the majority of surgeries performed by the gynecology and obstetrics department were obstetric in nature. Additionally, despite the high birth rates and repeated cesarean deliveries among migrant women, we observed a remarkably low number of sterilization procedures such as tubal ligation. We speculate that this reluctance may be related to a desire to preserve fertility in the face of war, as a means of ensuring familial and cultural continuity.

Differences in surgical needs among migrant populations may vary depending on the type of humanitarian crisis encountered, such as natural disasters or armed conflict.^31^ Ultimately, each crisis presents unique challenges, and surgical demands are context-specific. In our study, analysis of surgical procedures performed on migrant patients revealed that the three most frequently involved departments were orthopedics, gynecology, and obstetrics, and general surgery, in that order. This ranking was consistent with the data showing orthopedics and traumatology (20.63%), gynecology and obstetrics (17.51%), and general surgery (15.67%) as the most active departments.

It has been previously reported that approximately 5.7% of the refugee population in Türkiye has undergone surgery due to traumatic injuries.^32^ Similarly, a recent study by Cakmak et al.^33^ conducted in another regional hospital found that orthopedic surgeries were the most frequently performed procedures among Syrian refugees, with 59% related to extremity trauma. This high prevalence may be attributed to the inability of personal protective equipment to adequately shield limbs such as the arms and legs. Moreover, the nature of injuries sustained during wartime differs between civilians and military personnel. While soldiers are more commonly exposed to fatal penetrating injuries, civilians are more likely to experience blunt trauma, often as a result of environmental hazards or occupational accidents.^34, 35^ These patterns are reflected in our cohort, where orthopedic trauma surgeries constituted a significant proportion of operations among migrants.

Supporting this perspective, a study by Hornez et al.,^36^ which shared surgical management experiences from the Syrian conflict, reported that 69% of the most common cases involved extremity surgeries managed by orthopedic departments. Likewise, Biswas et al.^37^ in a study from Israel–another neighboring country affected by the Syrian crisis-also identified extremity-related orthopedic surgeries as the most frequently performed procedures on Syrian migrants during the war period. Taken together, these findings highlight the predominant need for orthopedic surgical care among war-affected populations, both in Türkiye and in other regions. Our study aligns with the existing literature by confirming that orthopedic surgeries-particularly those related to extremity injuries-were the most commonly performed procedures among migrant patients in our hospital. However, unlike the study by Cakmak et al.,^33^ which primarily focused on cost analysis, our study aimed to characterize the long-term surgical burden of migrants. This difference in focus may be considered a limitation of our study regarding economic evaluation.

When the general surgery procedures performed on migrants in our study were analyzed, emergency surgeries excluding appendectomy were found to be the most common interventions among Syrian patients, whereas appendectomy was more frequent among Turkish patients. This finding demonstrated that non-appendectomy emergency surgeries were significantly more frequent among Syrian migrants. Despite our hospital being located in a relatively small border province, the local population more than doubled within a year due to the influx of migrants. In the context of this significant demographic shift, it is important to note that prior research has suggested no racial or genetic differences in the incidence of appendicitis.^38^ Consistent with this, our findings revealed that the proportion of appendectomy cases for Syrian migrants was lower than among Turkish patients, supporting the notion that ethnicity does not significantly influence the incidence of acute appendicitis.^5^

Children, being in a rapidly developing stage of life, represent one of the most vulnerable age groups–especially in the context of war. In our study, 25.54% of the Syrian migrant patients were under the age of 18, indicating a significant paediatric presence among the surgical cases. This finding is consistent with the study by Bucak et al.^39^ While a previous study by Loucas et al.^40^ reported that tonsillectomy and circumcision were the most common surgical procedures among migrant children, our study found that non-emergency surgeries, other than circumcision, were the most frequently performed paediatric procedures among Syrian patients. This finding aligns with another study conducted in a Turkish border region, which similarly reported a predominance of non-traumatic elective surgeries among migrant children.^41^ Although due to our hospital’s proximity to the border, a higher rate of paediatric trauma surgeries might have been expected, we observed a relatively low rate of paediatric trauma cases, likely due to the referral of severe trauma patients to tertiary care centers with specialized services.

Although the American Academy of Otolaryngology has identified hearing loss, thyroid, and parathyroid disorders as the most common conditions requiring surgical intervention in low- and middle-income countries, a review of the literature reveals a lack of studies investigating the surgical needs of migrants within the field of otolaryngology (ENT).^42^ Existing data tend to focus on specific ENT-related conditions in migrant populations rather than their overall surgical burden.^43, 44^ In our study, the most frequently performed ENT procedures among Syrian migrants were tonsillectomy and adenoidectomy. Septorhinoplasty procedures were also performed, but at a lower frequency, reflecting a gradual shift towards quality-of-life-improving surgeries as integration into the host country progressed.

In times of crisis, both adults and children experience an increased need for ophthalmologic care, particularly in relation to visual impairments. One study highlighted that migrants have higher rates of visual impairment and blindness compared to host country populations.^45^ The same study also reported that cataract was the most common cause of vision loss among individuals aged 20 to 40 years. Consistent with these findings, our study identified cataract surgery as the most frequently performed ophthalmologic procedure among Syrian migrants over the past decade. Penetrating and sharp object injuries were found to be the second most common cause for ophthalmologic intervention in this population, which we attribute to war-related ocular trauma. Despite the long-term settlement of many migrants in host communities, inadequate nutrition may continue to contribute to the development of cataracts. It is well established that vitamin deficiencies can predispose individuals to cataract formation, and supplementation may help prevent or delay this condition.^46^

Due to the high kinetic energy of bullets and shrapnel during wartime, not only the directly affected organs but also adjacent tissues may sustain significant damage. Saleh et al.^32^ reported that 58% of trauma-related surgeries were due to injuries from bombs and firearms. Similarly, in a study by Cakmak et al.,^33^ intestinal injuries were found to be the most common outcome of gunshot wounds. Aras et al.,^47^ in their study involving 186 patients with firearm-related injuries, emphasized the critical importance of timely and effective surgical intervention. In a previous study conducted in our hospital during the peak of the Syrian civil war, Kocamer Şimşek et al.^48^ reported that the neurosurgery department had the highest mortality rate among all surgical units. In our study, the most frequently involved surgical specialties for patients operated under the “war code” were, in descending order, orthopedics, neurosurgery, plastic and reconstructive surgery, general surgery, and cardiovascular surgery. This distribution underscores the severity and complexity of war-related injuries and highlights the indispensable role of multidisciplinary surgical teams.

Given the complexity and severity of such cases, it is essential that border hospitals in countries with high capacity to treat this patient population are equipped not only with advanced medical infrastructure but also with highly trained and experienced personnel. The results of our study support this need. Among patients who underwent surgery under the war code, the mean age corresponded to 26-45 years, reflecting young adulthood; there was a statistically significant male predominance, with 91.59% of these patients being male, which is consistent with previous literature.^49, 50^ We believe this gender imbalance reflects the fact that many men remained in the conflict zones to fight, resulting in a higher proportion of male casualties requiring surgical care.

### Study Limitations

One of the main limitations of this study is its single-center design. As the data were derived from a single public hospital located on the Türkiye-Syria border, the findings may not be fully generalizable to other healthcare settings, regions, or populations with different demographic or institutional characteristics.

This study did not include an economic evaluation of the surgical services provided. As a result, the potential implications regarding the cost-effectiveness or cost-benefit of delivering such healthcare to migrant populations remain unexplored. Future studies incorporating economic assessments would be valuable to guide resource allocation and policy-making in similar high-demand, low-resource healthcare environments.

The most important limitation of our study is that it was conducted at a single tertiary care center, which may limit the generalizability of the findings. There is a pressing need for more comprehensive, multi-center studies evaluating surgical healthcare services provided to migrant and refugee populations in both secondary and tertiary hospitals. Although the retrospective design of our study is a limitation, its strengths include a large sample size, and the fact that, to our knowledge, no other study in Turkiye has addressed this topic in such depth.

In the future, prospective studies are warranted to better assess long-term surgical outcomes and postoperative complication rates. Such studies will contribute valuable data on access to surgical care and the quality of postoperative follow-up for migrant populations, ultimately guiding the improvement of healthcare delivery for displaced individuals.

## Conclusion

As a result of global migration movements, the prevalence of surgical conditions is notably high among refugee populations. However, a substantial proportion of displaced individuals are forced to seek medical care in countries with limited surgical capacity. Our study highlights the surgical burden imposed by war-related injuries and prolonged humanitarian crises on migrant populations. In this context, the effective management of surgical healthcare needs among migrants requires experienced medical teams, robust healthcare infrastructure, and a multidisciplinary approach within a well-organized system. For healthcare services in border-region hospitals to be more effectively planned and delivered, it is essential to maintain accurate patient records and analyze the distribution of surgical interventions. These data can help us better understand the surgical needs of migrant populations and serve as a foundation for developing responsive and sustainable health policies. Therefore, strategic efforts should be made to train healthcare professionals working in border areas and to strengthen hospital infrastructure in these high-demand regions. Finally, it is important to note that due to limitations in data accessibility, Turkish patient records for the first quarter of 2010 were unavailable, resulting in a three-month underrepresentation of surgeries performed on Turkish nationals.

## Ethics

**Ethics Committee Approval:** Ethical approval for the study was obtained from the Ethics Committee of Gaziantep University Faculty of Medicine (approval no: 2019/486, date: 05.02.2020).

**Informed Consent:** Ethics Committee due to the retrospective nature of the study.

## Figures and Tables

**Table 1. Demographics of Turkish and Syrian Patients:** 

**Category**	**Syrian n (%)**	**Turkish n (%)**	**Total n (%)**
Number of patients n (%)	22,124 (41.76)	30,854 (58.24)	52,978 (100)
**Gender**			
Gender - male n (%)	13,750 (62.15)	17,451 (56.57)	31,201 (59.89)
Gender - female n (%)	8,374 (37.85)	13,403 (43.43)	21,777 (41.11)
**Age**			
Mean age mean ± SD	28.90±18.45	32.98±21.42	31.28±20.33
Mean age - male mean ± SD	27.67±18.44	30.12±22.39	29.04±20.78
Mean age - female mean ± SD	30.92±18.29	36.71±19.46	34.48±19.23
**Age groups**			
0-17 n (%)	5,650 (25.54)	6,804 (22.06)	12,454 (23.51)
18-44 n (%)	12,434 (56.20)	15,596 (50.55)	28,030 (52.91)
45-65 n (%)	2,903 (13.12)	5,322 (17.25)	8,225 (15.53)
>65 n (%)	1,137 (5.14)	3,132 (10.14)	4,269 (8.06)

*n (%) - Mean ± SD, mean ± standard deviation

**Table 2. Distribution of Surgical Clinics Where Patients Received Services in the Last 10 Years by Ethnicity:** 

**Department of surgery**	**Syrian n (%)**	**Turkish n (%)**	**Total n (%)**
**Orthopedics and traumatology surgery**			
Large bone osteotomy and fixation	2,500 (22.87)	1,876 (17.17)	4,376 (40.04)
Other	2,882 (26.37)	3,671 (33.59)	6,553 (59.96)
Total	**5,382 (10.16)**	**5,547 (10.47)**	**10,929 (20.63)**
**Gynecology and obstetrics surgery**			
Caesarean section	3,483 (37.56)	4,888 (52.70)	8,371 (90.26)
Other	303 (3.27)	600 (6.47)	903 (9.74)
Total	**3,786 (7.15)**	**5,488 (10.36)**	**9,274 (17.51)**
**General surgery**			
Appendectomy	267 (3.21)	937 (11.29)	1,204 (14.50)
Emergency (excluding appendectomy)	595 (7.17)	319 (3.84)	914 (11.01)
Non-emergency	1,338 (16.12)	4,846 (58.37)	6,184 (74.49)
Total	**2,200 (4.15)**	**6,102 (11.52)**	**8,302 (15.67)**
**Urology**			
Endoscopic surgeries	620 (13.15)	899 (19.07)	1,519 (32.22)
Prostate and bladder surgeries	238 (5.05)	444 (9.42)	682 (14.47)
Varicocele-hydrocele-inguinal and testicular surgery	254 (5.39)	621 (13.17)	875 (18.56)
Circumcision	57 (1.21)	959 (20.34)	1,016 (21.55)
Others	298 (6.32)	324 (6.88)	622 (13.20)
Total	**1,467 (2.77)**	**3,247 (6.13)**	**4,714 (8.90)**
**Ear, nose and throat surgery**			
Emergency	84 (1.81)	81 (1.75)	165 (3.56)
Tonsil and adenoid surgeries	859 (18.50)	998 (21.50)	1,857 (40.00)
Septum and rhinoplasty surgeries	266 (5.73)	1,246 (26.84)	1,512 (32.57)
Others	331 (7.13)	777 (16.74)	1,108 (23.87)
Total	**1,540 (2.91)**	**3,102 (5.85)**	**4,642 (8.76)**
**Pediatric surgery**			
Emergency (excluding appendectomy)	188 (4.55)	59 (1.43)	247 (5.98)
Appendectomy	164 (3.97)	286 (6.93)	450 (10.90)
Circumcision	227 (5.50)	1,103 (26.70)	1,330 (32.30)
Non-emergency (excluding circumcision)	1,153 (27.92)	950 (23.00)	2,103 (50.92)
Total	**1,732 (3.27)**	**2,398 (4.53)**	**4,130 (7.80)**
**Plastic and reconstructive surgery**			
Skin and soft tissue surgeries	700 (22.22)	503 (15.96)	1,203 (38.18)
Others	568 (18.03)	1,066 (33.83)	1,634 (51.86)
Mandibula and maxilla surgery	262 (8.31)	52 (1.65)	314 (9.96)
Total	**1,530 (2.89)**	**1,621 (3.06)**	**3,151 (5.95)**
**Neurosurgery**			
Emergency	1,138 (39.34)	239 (8.26)	1,377 (47.60)
Non-emergency	732 (25.30)	784 (27.10)	1,516 (52.40)
Total	**1,870 (3.53)**	**1,023 (1.93)**	**2,893 (5.46)**
**Ophthalmology**			
Emergency	471 (16.80)	52 (1.85)	523 (18.65)
Non-emergency	909 (32.42)	1,372 (48.93)	2,281 (81.35)
Total	**1,380 (2.60)**	**1,424 (2.69)**	**2,804 (5.29)**
**Cardiovascular surgery**			
Emergency	563 (33.71)	133 (7.96)	696 (41.67)
Non-emergency	301 (18.02)	673 (40.31)	974 (58.33)
Total	**864 (1.63)**	**806 (1.52)**	**1,670 (3.15)**
**Thoracic surgery**			
Emergency	307 (65.46)	35 (7.46)	342 (72.92)
Non-emergency	66 (14.07)	61 (13.01)	127 (27.08)
Total	**373 (0.70)**	**96 (0.18)**	**469 (0.88)**

Total data are expressed as a percentage of the total number of cases compared to all operations.

Percentage distributions within departments were calculated separately

**Table 3. Surgical Distribution of Immigrants Operated with War Code:** 

**Department of surgery**	**Number (n)**	**Percentage (%)**
Orthopedics and traumatology surgery	1739	34.70
Neurosurgery	811	16.18
Plastic and reconstructive surgery	792	15.81
General surgery	474	9.46
Cardiovascular surgery	403	8.04
Ophthalmology	353	7.04
Thoracic surgery	262	5.23
Pediatric surgery	99	1.98
Ear, nose and throat surgery	50	1.00
Urology	26	0.52
Gynecology and obstetrics surgery	2	0.04
